# The capability of heterogeneous γδ T cells in cancer treatment

**DOI:** 10.3389/fimmu.2023.1285801

**Published:** 2023-11-24

**Authors:** Wenyi Yan, Louisa S. Chard Dunmall, Nicholas R. Lemoine, Yaohe Wang, Yafeng Wang, Pengju Wang

**Affiliations:** ^1^ Sino-British Research Centre for Molecular Oncology, National Centre for International Research in Cell and Gene Therapy, State Key Laboratory of Esophageal Cancer Prevention & Treatment, School of Basic Medical Sciences, Academy of Medical Sciences, Zhengzhou University, Zhengzhou, China; ^2^ Centre for Biomarkers & Biotherapeutics, Barts Cancer Institute, Queen Mary University of London, London, United Kingdom; ^3^ State Key Laboratory of Esophageal Cancer Prevention & Treatment, School of Pharmaceutical Sciences, Zhengzhou University, Zhengzhou, China

**Keywords:** γδ T cells, T cell subsets, heterogeneity, CAR-γδ T, adoptive cell transfer therapy, cancer immunotherapy

## Abstract

γδ T cells, a specialized subset of T lymphocytes, have garnered significant attention within the realm of cancer immunotherapy. Operating at the nexus between adaptive and innate immunological paradigms, these cells showcase a profound tumor discernment repertoire, hinting at novel immunotherapeutic strategies. Significantly, these cells possess the capability to directly identify and eliminate tumor cells without reliance on HLA-antigen presentation. Furthermore, γδ T cells have the faculty to present tumor antigens to αβ T cells, amplifying their anti-tumoral efficacy.Within the diverse and heterogeneous subpopulations of γδ T cells, distinct immune functionalities emerge, manifesting either anti-tumor or pro-tumor roles within the tumor microenvironment. Grasping and strategically harnessing these heterogeneous γδ T cell cohorts is pivotal to their integration in tumor-specific immunotherapeutic modalities. The aim of this review is to describe the heterogeneity of the γδ T cell lineage and the functional plasticity it generates in the treatment of malignant tumors. This review endeavors to elucidate the intricate heterogeneity inherent to the γδ T cell lineage, the consequential functional dynamics in combating malignancies, the latest advancements from clinical trials, and the evolving landscape of γδ T cell-based oncological interventions, while addressing the challenges impeding the field.

## 1 Introduction

There is a distinct and conserved population of T lymphocytes called γδ T cells, named for the γ and δ chains making up the T cell receptor (TCR) that sets them apart from the classical T cells (CD4^+^ and CD8^+^) that contain αβ TCRs. They represent a distinctive subset of T cells that exist in a transitional state between the adaptive and innate immune systems (1–3). Functionally, there is compelling evidence to suggest that their antigen receptors exhibit greater specificity and diversity compared to the surface antigen receptors found in αβ T cells or B cells (4). γδ T cells play an important role as the first line of defense of the immune system while also participating in the adaptive immune response. Serving as a conduit between innate and adaptive immunity to elicit potent reactions (5), γδ T cells are viewed as promising immunotherapeutic agents within the realm of cancer treatment, offering a fresh perspective in the field of anti-tumor immunity (6, 7).

## 2 Development and differentiation of human γδ T cells

In many mammalian species, γδ T cells emerge as the primary lymphocyte subset during fetal development (8, 9). Their receptor, composed of a γ and a δ chain, is formed through somatic variable-diversity-joining (V(D)J) recombination, similar to the segments of α- and β-chains in αβ TCRs (10). There are numerous configurations for the T cell receptor’s γδ variable region (Vγ) and delta chain variable region (Vδ), and the fusion of these two regions allows for the formation of a sizable collection of roughly 10^20^ TCR clonotypes (11), providing significant diversity to γδ T cell subsets.Human γδ T cells are traditionally classified into three primary subgroups: Vδ1, Vδ2, and Vδ3, determined by the Vδ chain usage (12–14). Among the three main γδ T cell subsets in humans, Vδ1 T cells predominantly pair with the Vγ I family, which includes (Vγ2/3/4/5/8), and the Vδ2 subset predominantly binds Vγ II (Vγ9), typically Vγ9Vδ2 T cells (15). Unique tissue localization, activation, and function are displayed by various γδ T cell subsets and their distribution within the human body can be distinguished clearly (16–19). Vδ1 T cells are predominantly located in epithelial tissues, including the intestines and skin, as well as organs like the spleen and liver. These cells have a vital function in safeguarding the preservation of epithelial tissue integrity (20). Vδ2 T cells, mainly the Vγ9Vδ2T cell subset, account for approximately 60-95% of peripheral γδ T cells in the circulation (21). These cells make up around 2-5% of the circulating CD3^+^ T cell population and play a dual role as both effector cells and antigen-presenting cells (APCs) (22). Vδ3 T cells, which are infrequently observed in circulatory systems, are notably prevalent in hepatic tissues, particularly in individuals with infections or malignancies.

## 3 γδ T cell in complex tumor microenvironments

### 3.1 Recruitment of γδ T cells to the tumor microenvironment

The tumor microenvironment (TME) significantly influences the activity of γδ T cells across various cancers. In the complex TME, γδ T cells are recruited or activated toward the tumor site. However, there also exists a synergistic or pleiotropic effect of tumor cells and multiple factors in the TME, where infiltrating γδ T cells are activated or depleted, or polarized to a tumor-promoting phenotype, thus supporting cancer progression (18).

Some investigators have analyzed the effect of the TME on γδ T cell recruitment in a preclinical transplantable B16 melanoma model, where human Vδ1 T cells use the CCR2/CCL2 pathway to migrate toward the tumor, where they exert critical non-redundant anti-tumor functions (23). Consistent with this study, Vδ1 T cell infiltration was abundant in breast and primary prostate cancers with significantly upregulated CCL2 expression (24, 25). Furthermore, in cases of hepatocellular carcinoma (HCC), tumor cells harness the CCL4/CCL5 chemokine pathway, interacting with the CCR1/CCR5 receptors, thereby orchestrating the mobilization of γδ T cells either from the peripheral blood or peritumor region to the tumor region (26). In the TME of breast cancer, breast cancer cells secrete IP-10, which mediates the transport and migration of γδ1 T cells to the tumor site via IP-10/CXCR3 (27, 28). It has also been claimed that the CCR4/CCR8-CCL17/CCL22 pathway also significantly induces Vδ1 T cell migration. Meanwhile, high levels of CCL17 and CCL22 were detected in a variety of tumors, such as lung cancer, gastric cancer, B-cell non-Hodgkin’s lymphoma, Hodgkin’s lymphoma, and peripheral T-cell lymphoma. In lymphomas, CCL17 was specifically expressed in classical Hodgkin’s lymphoma, whereas CCL22 was expressed in nodular lymphocyte-predominant Hodgkin’s lymphoma and B-cell non-Hodgkin’s lymphoma (29).

### 3.2 Heterogeneity of γδ T cells in the tumor microenvironment

Both *in vivo* and *in vitro* studies have revealed the multifaceted roles of various γδ T cell subtypes in modulating tumor cell proliferation, underscoring their intricate contribution to the dynamics of cancer progression. Flow cytometry and transcriptome analyses revealed that tumor-infiltrating lymphocytes contained an average of 4% γδ T cells, most of which expressed Vδ1. Among γδ T cells in the TME, the Vδ1 T cell subset highly expresses CXCR1 and weakly expresses CCR5, whereas Vγ9Vδ2 T cells show only strong expression of CCR5 (30). Moreover, Vγ9Vδ2 T cells concurrently expressed CCR3 and CXCR3, enabling them to initiate anti-tumoral responses in peripheral tissues, especially during the metastatic processes (18).

Vγ9Vδ2 T lymphocytes have been identified to demonstrate cytotoxic properties against breast cancer cells, enhancing apoptotic pathways and attenuating angiogenic signaling processes (31). Accumulated γδ1 T cells in the breast TME are termed γδ1 Tregs (32, 33), and these breast tumor-derived γδ Tregs suppress innate and adaptive immunity by inducing immune senescence and preventing dendritic cell maturation and activity (24, 34).

In the study of γδ T cells in the TME of colorectal cancer (CRC), the results showed that γδ T cells were mainly detected in paracancerous tissues but rarely in intra-tumoral tissues, and there was no significant increase in the number of T cell subpopulations of Vδ1 and Vδ2 in the CRC-infiltrating γδ T cells, but the main subpopulation was Vδ1 T cells (35, 36). The shifted balance between these subpopulations might hold implications for the progression of colon cancer (37).

Transcriptomic analysis of the peripheral blood of leukemia patients showed the presence of many tumor-infiltrating Vγ9Vδ2 cells, which positively correlated with the survival of these patients (18, 38). But then a new finding emerged that patients with chronic lymphocytic leukemia (CLL) had an increased percentage of Vδ1 cells, which replaced Vγ9Vδ2 cells as the predominant γδ T-cell subtype in the peripheral blood (39). And the study noted that a higher percentage of Vγ9Vδ2 cells was associated with a poor prognosis in patients with untreated CLL, as these lymphocytes exhibited signs of functional failure with reduced NKG2D expression (40, 41).

Infusion of large numbers of γδ T cells (Vδ1 and Vδ2 T cells) into high-risk leukemia patients by allogeneic hematopoietic stem cell transplantation (HSCT) contributes to the rapid control of infections and leukemia relapse. In HSCT recipients, Vδ2 and Vδ1 T cells were found to be cytotoxic to primary acute leukemia cells, whereas newly generated Vδ1 and Vδ3 cells in the TME underwent an adaptive response driven by cytomegalovirus (CMV) reactivation (42).

## 4 γδ T cells funtional flexibility

Despite accounting for a relatively small proportion of total T cells, γδ T cells have a complex and crucial role in the onset and progression of cancer. The function of γδ T cells in the TME can be altered by several circumstances to become either support tumor growth or combat it. Subsets of γδ T cells indirectly achieve anti-tumor immunity by producing specific factors to promote Th1, Th2, or Th17 differentiation (43–45) or cross-transmitting signals with B cells (46, 47), natural killer (NK) cells (45), and dendritic cells (48) in TME (49). There are also specific subpopulations of γδ T cells secrete a quantity of IL-17, which can directly act on epithelial cells to promote the progression of cancer, and γδ T can affect αβ T cells through immune checkpoints, supporting the creation of an immunosuppressive microenvironment that promotes tumorigenesis (2, 50). This dual role may be attributed to the inherent plasticity of γδ T cells, which includes the recruitment or residence of specific γδ T cell subsets at the tumor site and the ability to differentiate into different functional cell subsets based on the TME (51, 52).

### 4.1 Anti-tumor function

In the realm of oncology, γδ T cells serve as a robustly positive prognostic indicator in most malignancies (47, 53, 54). Pan-cancer analysis based on the TCGA database in 2015 showed that γδ T cells were the best predictor of the prognosis within a range of solid tumors (50). γδ T cells are crucial for cancer immune surveillance and indeed studies have found that the incidence of cancers in mice lacking γδ T cells increases (55). Notably, γδ T cells accumulate in tumor-associated lymphoid tissues (38, 56) and can penetrate solid tumor tissues (57, 58). They can naturally infiltrate into the tissues of the whole body, including the lung, liver, and intestinal tract, which can be difficult malignancies to penetrate therapeutically.

The γδ TCR of Vγ9Vδ2 T cells is highly sensitive to tumor perception. During the course of tumorigenesis, the intracellular accumulation of phosphoantigens (pAgs) such as isoprenyl diphosphate (IPP) and dimethylallyl diphosphate (DMAPP) can weakly activate these cells. Meanwhile, the exogenous pAg (E)-4-hydroxy-3-methyl-but-2-enyl pyrophosphate (HMB-PP) can be co-locked intracellularly by transmembrane chymotrypsin 3A1 (butyrophilin 3A1, BTN3A1) and BTN2A1, and extracellularly detached and bound to the γδ TCR, resulting in efficient activation of γδ T cells (59, 60).

**Figure 1:**
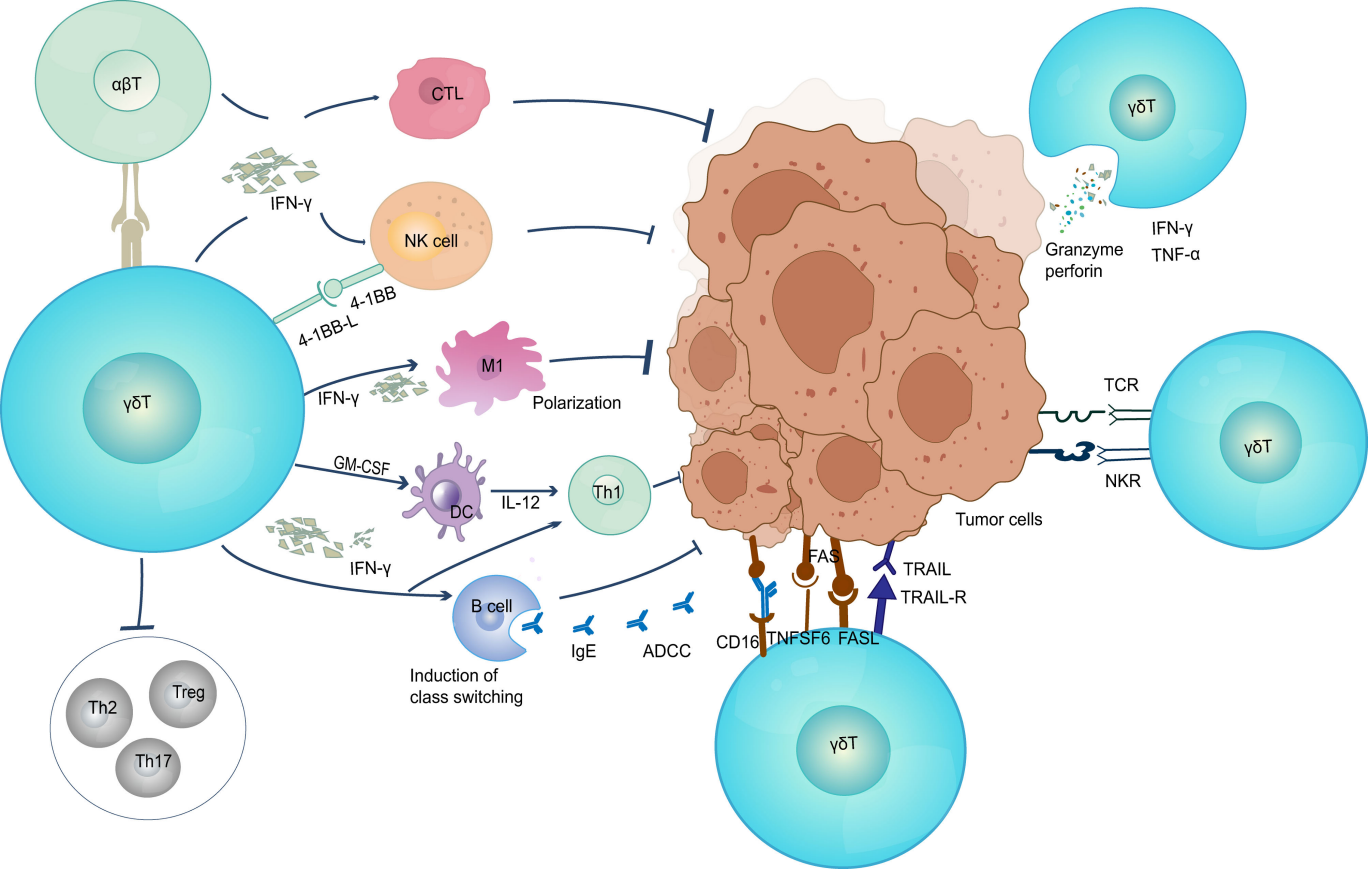
The anti-tumor function of γδ T cells. γδ T cells elicit antitumor immune responses through multiple pathways (1) Direct killing effect; (2) Secretion of IFN-γ and TNF-α; (3) Induced B cell transformation to secrete large amounts of Ig E and produce adaptive immune responses; (4) Eliciting CD8^+^ T cell responses. TNF, tumor necrosis factor; TRAIL, TNF-related apoptosis-inducing ligand; ADCC, antibody-dependent cell-mediated cytotoxicity; CTL, cytotoxic T lymphocyte; NK, natural killer cell; DC, dendritic cells.

γδ T cells have characteristics of both the innate and adaptive immune systems and can act directly or indirectly on tumor cells (**Figure 1**; **Table 1**). To directly attack cells, γδ T cells rapidly migrate into the local tumor microenvironment by recognizing NK cell receptors on the cell surface. γδ 1T cells and γδ 2 T cells are both capable of ex vivo lysing of tumor cells and express chemokine receptors that enhance tumor homing (4). Activated γδ T cells can release granzyme and perforin to kill tumor cells directly (78). In addition, different γδ T cell subsets attach to tumor cells through the death receptors TNF-related apoptosis-inducing ligand receptor (TRAILR), CD95 (also known as FAS), and TRAIL and lyse cancer cells (65, 79, 80). The cell surface receptors NKG2D (81) and CD16 (48) also mediate the direct killing of γδ T cells based on antibody-dependent cytotoxicity and effector responses (48, 82). Complementing their cytotoxic capabilities, γδ T cells can also secrete cytokines IFN-γ and TNF-α, jointly suppressing tumor-associated angiogenesis (83).In some hematologic tumors, γδ T cells have been found to be capable of immunosurveillance by NK-like mechanisms (81, 84). Remarkably, around 80% of quiescent circulating γδ T cells express NK receptors. Most of these cytotoxic Vγ9Vδ2 T cell clones express HLA class I inhibitory NK cell receptors, such as CD94/NKG2A, KIR2DL1, KIR2DL2, KIR3DL1, or KIR3DL2 (85). Intriguingly, the majority of the clones express several different receptors, which help them to recognize different types of tumors (86). In breast cancer, Vδ1T cells residing at the tumor site recognize the tumor through innate stimuli including NKG2D (87). Within the TMEs, γδ T cells exert intermediate anti-tumor effects by interacting with B cells, dendritic cells, αβ T cells, and NK cells. γδ T cells can be used as antigen-presenting cell to activate αβ T cells (68). They can increase the amount of IFN-γ secreted by αβ T to regulate the TME by inducing recruitment of CTL, NK cells, and Th1, inducing M1-type polarization of macrophages (12), activating dendritic cells to induce their maturation (88), upregulating the expression of MHC class I in tumor cells to improve anti-tumor immune response (89) and preventing pro-tumor T helper cells from functioning (Treg, Th17 and/or Th2). Additionally, epithelial Vγ5 T cells induce B cell transformation and secretion of large amounts of Ig E, CCR5-expressing Vγ9Vδ2 T cell subsets promote antibody production and class switching (90), leading to the development of an immediate adaptive immune response in skin malignancies brought on by chemicals (91).

### 4.2 Pro-tumor function

**Figure 2:**
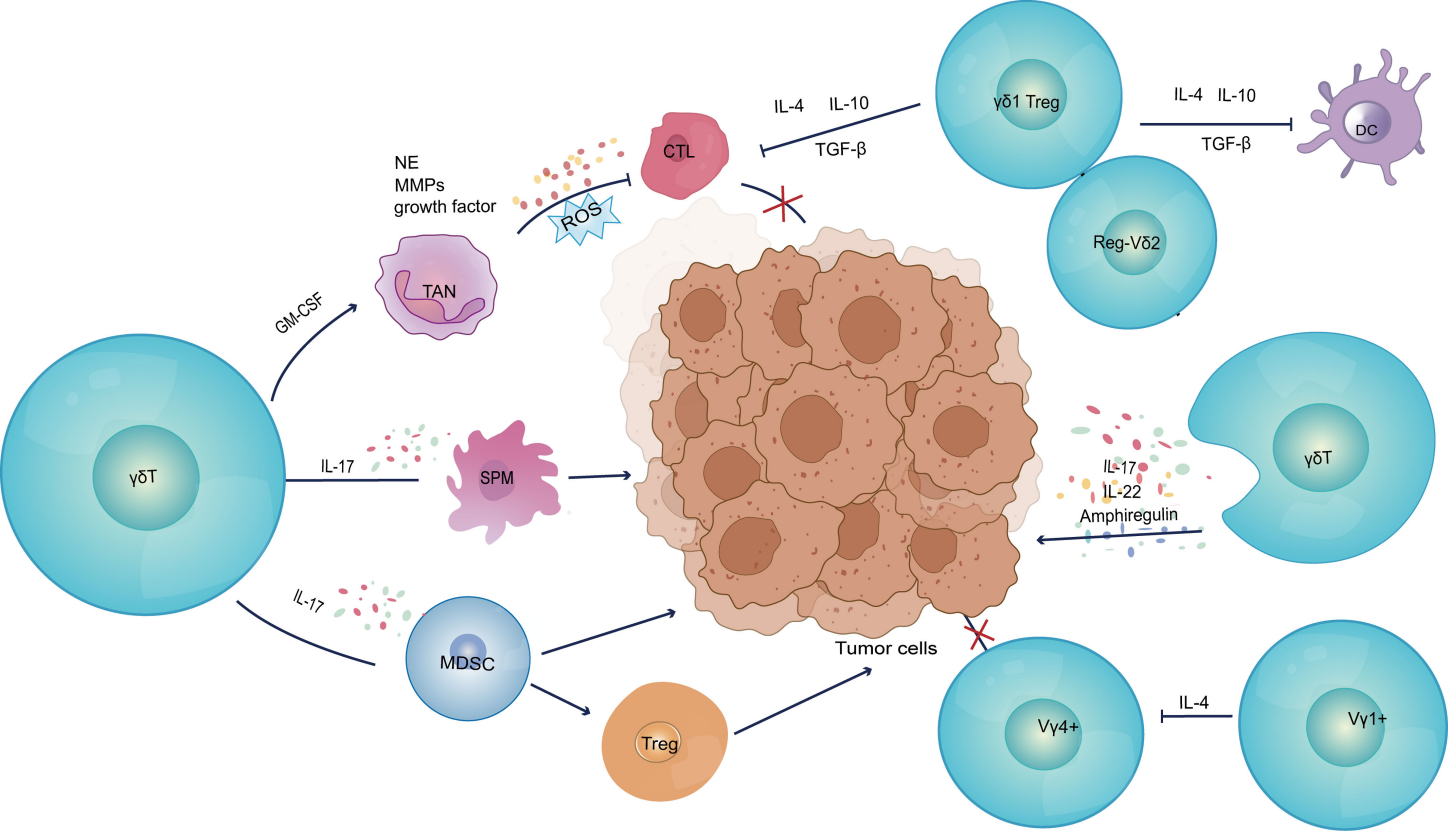
Pro-tumor functions of γδ T cells. (1) Secretion of IL-17 induces tumor cells to express pro-angiogenic factors, as well as mobilizing SPM to promote inflammatory response and angiogenesis. (2) Inhibition of anti-tumor immune response; (3) Construction of an immunosuppressive microenvironment; (4) Secretion of IL-17 to upregulate AM and endothelial cell permeability, as well as production of IL-22 and AREG to directly induce tumor cell proliferation. VEGFA, vascular endothelial growth factor A; ANG2, angiopoietin-2; SPM, small peritoneal macrophages; MDSC, myeloid-derived suppressor cells; TAN, tumor-associated neutrophils; G-CSF, granulocyte colony-stimulating factor; NE, neutrophil elastase; MMPs, matrix metalloproteinases; AM, adhesion molecule.

Specific γδ T cells within the tumor microenvironment are known to secrete IL-17 (92), which promotes the emergence of autoimmune and inflammatory disorders (93, 94). At the same time, IL-17-producing γδ T cells promote the growth of tumors in a variety of ways. Recent studies have elucidated five key characteristics that underscore the tumor-promoting roles of these γδ T cells (**Figure 2**; **Table 1**). Firstly, γδ T cells have been shown to have a pro-angiogenic effect (95). Vascular endothelial growth factor (VEGF) and angiopoietin-2 (ANG-2) are angiogenic factors that γδ T cells can produce to promote angiogenesis (92, 96). Moreover, Margarida Rei et al. discovered that small peritoneal macrophages (SPM) were activated by IL-17-secreting Vγ6 γδ T cells, which accelerated the progression of ovarian cancer. Migration inhibitory factor (MIF) and IL-6 are two of the many tumor-promoting mediators that SPM can generate. They may promote the development of a variety of pro-inflammatory and pro-angiogenic molecules, while also protecting tumor cells from death (96). Secondly, these cells can prevent immune cells from performing their anti-tumor immunological functions. Specifically, IL-17 production from γδ T cells can directly suppress the anti-tumor activities of CTL and Th1 cells. Additionally, a significant proportion of γδ1 Treg cells can be found in the human breast tumor microenvironment, and they exert potent inhibitory effects on the proliferation of CD4^+^, CD8^+^, and Vγ9Vδ2 T cells by inducing senescence in responding immune cells and impairing the maturation and function of DCs (24, 27, 32). Elevated BMP2 in Acute Myeloid Leukemia (AML) patients induces the production of CD25^+^CD127^low^Vδ2^+^ T cells (named Reg-Vδ2). Reg-Vδ2 cells produce a number of regulatory cytokines rather than inflammatory cytokines, and the anti-AML activity of effector Vδ2 cells is significantly inhibited by Reg-Vδ2 cells (97). Furthermore, Vγ1 γδ T cells secrete IL-4 and decrease the NKG2D, perforin, and interferon expression levels in Vγ4 γδ T cells (76).

**Table 1:** Mechanisms of γδ T cell effects on tumors.

Mechanism	γδ T cell subsets	Cancer cell type	Ref.
Anti-tumor			
perforin and granzyme B secretion to induce cytotoxicity	Vδ1 T cells	primary multiple myeloma cells	(61)
	Vγ9Vδ2 T	renal cell carcinoma	(62)
	CD56^+^ γδ T-cell	squamous cell carcinoma	(63)
kill tumor cells via trans-antibody dependent cell mediated cytotoxicity (ADCC)	Vγ9Vδ2 T	breast cancer	(64)
FasL- and TRAILR-mediated apoptosis of tumor cells	γδ T cells	PDAC	(65)
	γδ T cells	lung cancer cell lines	(66)
cross-present tumor antigens and stimulate αβ T cell activation and proliferation	Vγ9Vδ2 T	breast cancer stem-like cells	(67–69)
co-stimulate NK cells via 4-1BB	γδ T cells	squamous cell carcinoma head and neck tumor cell lines	(70)
Pro-tumor			
promote angiogenesis and tumour cell proliferation	IL17 producing γδ T cells	gallbladder cancer, hepatocellular carcinoma	(71, 72)
mobilizes neutrophils and polymorphonuclear myeloid- derived suppressor cells (PMN-MDSCs)	IL17 producing γδ T cells	colorectal cancer, hepatocellular carcinoma	(73, 74)
inhibit the activity of cytotoxic CD8^+^ T cell	IL17 producing γδ T cells	pancreatic cancer	(75)
Pro-tumor			
regulatory in Vγ4-mediated tumor immunity	Vγ1 γδ T cells	mouse melanoma	(76, 77)
suppress the activity of αβ T cells and dendritic cells through induction of senescence	Vγ1 γδ T cells	breast cancer	(24, 32)

CG, control group; IQR, interquartile range; MRD, measurable residual disease.

Thirdly, γδ T cells can directly construct a tumor immunosuppressive microenvironment. In human colorectal cancer, γδ T cells are polarized by microorganisms present due to disruption of the tumor epithelial barrier and inflammatory dendritic cells (Inf-DCs) in the TME to produce cytokines such as TNF-α, GM-CSF, IL-17 and IL-8. These cytokines recruit myeloid derived suppressor cells (MDSC) into the TME, regulate the development of tumor cells as well as induce Treg differentiation (73). In addition, these cells encourage G-CSF-mediated tumor-associated neutrophils (TAN) proliferation and accumulation in the TME. These TAN, in turn, release a variety of cancer-promoting factors, such as growth factors, neutrophil elastase (NE) and metalloproteinases (MMPs), and produce reactive oxygen species (ROS). These actions promote development of an immunosuppressive tumor microenvironment, inducing the depletion of CD8^+^ T cells and supporting tumor metastasis, tumor growth and invasion (31). Zhang et al. demonstrated that the “γδT cell-IL17A-Neutrophil” axis in the breast cancer tumor microenvironment promotes immunosuppression as well as enhancing the breast cancer’s tolerance to high-dose anti-VEGFR2 therapy (98).

In addition to this, there are two important protumor mechanisms, IL-17 secreted by γδ T cells modulates adhesion molecules and upregulates endothelial cell permeability to promote tumor metastasis (99). γδ T cells also produce IL-22 and amphiregulin (AREG), which directly induced tumor cell proliferation (100).

## 5 γδ T cell-based cancer therapy

γδ T cells can directly identify and kill tumor cells therefore, adoptive and *in vivo*-induced γδ T cell expansion therapies are promising avenues to explore for anti-cancer immunotherapy purposes (101, 102). γδ T cells may be more favourable for use in adoptive cell immunotherapy compared to αβ T cells as they react more quickly to targets to produce effector factors, and they are found in a range of organs (103). In the hypoxic tumor microenvironment, γδ T cells, particularly the Vδ 1 subset, exhibit greater tissue tendency and greater invasiveness compared to αβ T cells (104). Moreover, graft versus host disease (GvHD) and allogeneic response risks can be decreased by using γδ T cells’ MHC-independent identification of target cells (105). Currently, various strategies are being used to activate and target γδ T cells, including drugs, antibodies, and genetic engineering. These strategies aim to enhance the anti-tumor response of γδ T cells and use them to combat hematological or solid tumors, such as B-cell malignancies (106).

### 5.1 CAR-γδ T

CAR-T cell therapy is a type of immunotherapy that uses the patient’s own immune cells to fight cancer. In this approach,T cells are collected from the patient’s blood and genetically modified in the laboratory to express chimeric antigen receptors (CARs) on their surface. These CARs are designed to recognize specific proteins, called antigens, on the surface of cancer cells. Once the T cells have been modified, they are grown in large numbers and infused back into the patient’s body. The CAR-T cells can then seek out and destroy cancer cells that express the target antigen (107).

In the context of CAR-γδ T cells, diverse extracellular and intracellular domains can be fashioned based on the target antigen, the required co-stimulatory signal, and the signaling partner (108). Some examples of CAR designs for γδ T cells are: CD19-CAR, GD2-CAR (109), CD20-CAR (110), NKG2D-CAR (111), CCR (chimeric co-stimulatory receptor) (112), and NSCAR (non-signaling CAR) (113). To generate CAR γδ T cells, different methods of delivering genes can be used, for example, retrovirus (114), lentivirus (115), transposon (116), or mRNA electroporation (117). Traditional CAR-αβ T cell therapy has produced good clinical data in leukemia and other hematological malignancies, but it has not achieved the same success in solid cancer. In this regard, CAR-γδ T cells might offer a more promising avenue, as they have innate cytotoxicity capabilities, can recognize multiple antigens (118) and acquire the phenotypic and functional properties of antigen-presenting cells (APCs) (59, 119). In preclinical studies, CAR γδ T cells have exhibited potential against a diverse range of hematological and solid tumors, including B-cell lymphoma (110), glioblastoma (120), melanoma (121), colorectal cancer, and ovarian cancer (111). Nevertheless, several challenges persist in the development and application of CAR γδ T cell therapy (122), such as reduced tumor-toxicity, homing, *in vivo* persistence, heterogeneity, inter-donor variability, tumor microenvironment adaptation, etc.

### 5.2 Adoptive transfer and *In vivo* expansion of γδ T cells

Adoptive transfer of γδ T cells is a form of cancer treatment that involves the infusion of a patient’s own γδ T cells that have been expanded and activated outside the body. Nevertheless, previous clinical trials utilizing autologous γδ T cells sourced from cancer patients have only demonstrated limited clinical efficacy (123). Hence, current research is increasingly focusing on adoptive transfer therapies with allogeneic Vγ9Vδ2 T cells (124), which have been shown to enhance immune function, including CD4^+^ T cell, CD8^+^ T cell, and NK cell counts in cancer patients, even leading to total remission of recurrent hepatocellular carcinoma in one notable case (125). Another study investigated the use of adoptive cell therapy with IL-15-induced γδT cells in a patient-derived renal cell carcinoma xenograft model. The study concluded that IL-15-induced γδ T cells effectively suppressed tumor growth *in vivo* and prolonged the survival time of RCC-bearing patient−derived xenograft (PDX) mice (126).

*In vivo* expansion of γδ T-cells stands out as a unique approach to cancer immunotherapy. Unlike the ex vivo expansion seen in adoptive transfer, this method seeks to stimulate γδ T-cells directly within the patient’s body. This approach aims to enhance the anti-tumor activity of γδ T cells by using agents such as zoledronate, phosphoantigens, or specific cytokines such as IL-15 or IL-2 (124). *In vivo* expansion of γδ T cells has been shown to induce tumor regression and prolong survival in some animal models and clinical trials. A pilot study evaluated the adoptive transfer and *in vivo* expansion of haploidentical γδ T cells in patients with advanced hematological malignancies ineligible for allogeneic transplantation. Patients received peripheral blood mononuclear cells from half-matched family donors, followed by zoledronate and IL-2 to stimulate donor γδ T cells *in vivo*. This resulted in significant expansion of donor γδ T cells, NK cells, and double-negative αβ T cells. Impressively, three out of four patients achieved complete remission despite prior refractoriness (127).

## 6 Clinical trials: current state of the art

At present, many preclinical studies have been conducted by researchers that suggest that γδ T cell therapy works well in multiple tumor models. While treating advanced ovarian cancer, γδ T cells function in the patient’s ascites and tumor by innate and adaptive immunological methods, respectively. This may make γδ T cells a viable treatment option for advanced ovarian cancer (128). For hematological malignancies, researchers have explored various ways to treat tumors using γδ T therapy. Ganesan et al. created a Vγ9/CD123 bispecific antibody that specifically triggers Vγ9^+^ γδ T cells and causes cytotoxicity to the tumor *in vitro*. This antibody efficiently induces Vγ9^+^ γδ T cells to engage with tumor cells. In patients with AML, these cells possess a variety of strategies for mounting an efficient immune response against overloaded tumor cells (129). γδ T cells can identify cancer antigens other than peptides, so extending the pool of possible targets for tumor cell eradication. Combining this feature, Xu et al. proposed a new TCR-T platform. They designed the AbTCR with non-MHC-restricted targets like CD19, which allows for the management of cytokine-related toxicity beyond existing anti-CD19 CAR-T therapies and provides comparable tumor suppression (115). Contrary to conventional CD19 CAR-αβ T, CAR-γδ T cells may still be able to target leukemia cells that lack the CD19 antigen and as such are useful for cases in which the antigen has been lost (130).

Early clinical results have established the promising vista of γδ T cells therapies in leukemia and other hematological tumors and solid tumors such as lung, gastric, and liver cancers. A landmark study led by Zhinan Yin’s team monitored patients with advanced liver and lung cancers over three years post-reception of allogeneic γδ T cell therapy. The team used allogeneic Vγ9Vδ2 γδ T cells from healthy human sources. By treating 132 patients with advanced lung and liver cancer tumors with a total of 414 cell transfusions, their study found that there was not a single case of serious side effects from the allogeneic γδ T cell transfusions and only some patients developed transient, mild clinical reactions (125, 131). Furthermore, the results highlighted a significant extension in survival among eight liver cancer patients and ten lung cancer patients who received ≥5 cell infusions (130). During this decade, dozens of clinical trials have been approved and several products have emerged as well (132). Numerous biotechnological enterprises are channeling significant investments into this burgeoning domain (**Table 2**). The γδ T treatment proposed by French biologics company ImCheck Therapeutics comprises a novel human-derived anti-BTN3A antibody, ICT01. ICT01 is a monoclonal antibody that specifically promotes Vγ9Vδ2 T cells targeting of BTN3A, which is extensively expressed in diverse solid and hematologic malignancies. ICT01 has been shown to have antitumor activity *in vitro* and *in vivo* tumor models against a range of cancers. The study published preliminary data from the first phase 1/2a clinical study on ICT01, revealing the value of the potential clinical application of ICT01 in the care of people with developing malignancies (133).

**Table 2:** Clinical attempts at tumor immunotherapy using γδ T cells.

Sponsor	Product name	Treatment strategy	Target	Indications	Phase	Clinical Registration
Adicet Bio	ADI-001	CAR-γδ T cells	CD20	B cell lymphoma	Phase 1	NCT04735471, (110)
CytoMed Therapeutics	CTM-N2D	allogeneic NKG2DL-targeting CAR γδ T Cells	NKG2DL	Advanced Cancers	Phase 1	NCT05302037
CytoMed Therapeutics	CTM-N2D	Haplo/NKG2DL-targeting CAR γδ T Cells	NKG2DL	Solid tumors	Phase 1	NCT04107142, (117)
Beijing Doing Biomedical Technology	Anti-CD19-CAR γδ T cells	CAR-γδ T cells	CD19	B cell lymphoma, ALL, CLL	phase 1	NCT02656147
PersonGen BioTherapeutics	modified CAR -γδ T cells	CAR-γδT cells	CD7	relapsed or refractory CD7^+^ T cell-derived malignancies	Early Phase 1	NCT04702841
Lava Therapeutics	LAVA-051	γδ bsTCE	CD1d	CLL, MM, AML	Phase 2	NCT04887259, (133)
Lava Therapeutics	LAVA-1207	γδ bsTCE	PSMA	mCRPC	Phase 2	NCT05369000
ImCheck Therapeutics	ICT01	activator Vγ9Vδ2T cells	BTN3A (CD277)	solid tumors, blood cancers	Phase 2a	NCT04243499 NCT05307874, (131)
Gadeta BV	TEG002	autologous T cells transduced with a specific γδ TCR	HLA	MM, ovarian cancer	phase 1	NCT04688853, (134)
Peking University	ET190L1 ARTEMIS™ cell	AbTCR-T platform	CD19	B cell lymphoma	Phase 1	NCT03415399, (129)
IN8bio	INB-100	expanded/activated γδ T cell infusion	—	leukemia	Phase 1	NCT03533816
IN8bio	INB-200	gene-modified autologous γδ T cells	—	Glioblastoma	Phase 1	NCT04165941, (135)
TC BioPharm	ImmuniCell®	autologous γδ T cells	—	Malignant Melanoma, RCC, NSCLC	Phase 2	NCT02459067
TC BioPharm	OmnImmune®	allogeneic γδ T Cell therapy	—	AML	Phase 2b/3	NCT05358808
GammaDelta Therapeutics	GDX012	allogeneic Vδ1 T Cell therapy	—	AML	Phase 1	NCT05001451, (87, 136)
Acepodia Biotech	ACE1831	allogeneic γδ T Cell therapy	CD20	B-NHL	Phase 1	NCT05653271
302 Military Hospital of China	Allogeneic γδ T cells	allogeneic γδ T Cell therapy	—	HCC	Phase 1	NCT04518774
Emory University	Allogeneic Expanded γδ T	allogeneic Expanded γδ T Cells with GD2 Chemoimmunotherapy	GD2	neuroblastoma	Phase 1	NCT05400603
Chinese PLA General Hospital Medical School of Chinese PLA	ex vivo expanded allogeneic γδ T cells	allogeneic γδ T Cell therapy	—	Hematological Malignancies	Phase 2	NCT04764513
Chinese PLA General Hospital Medical School of Chinese PLA	ex vivo expanded allogeneic γδ T cells	allogeneic γδ T Cell therapy	—	Solid Tumors	Phase 2	NCT04765462
Wuhan Union Hospital, China	Ex-vivo expanded γδ T cells	allogeneic γδ T Cell therapy	—	AML	Phase 1	NCT04008381
Fuda Cancer Hospital	Vγ9Vδ2T	allogeneic Vγ9Vδ2T	—	Lung Cancer	Phase 2	NCT03183232, (130)
Fuda Cancer Hospital	Vγ9Vδ2T	allogeneic Vγ9Vδ2T	—	Pancreatic Cancer	Phase 2	NCT03180437
Fuda Cancer Hospital	Vγ9Vδ2T	allogeneic Vγ9Vδ2T	—	NSCLC	Phase 2	NCT02425748
Fuda Cancer Hospital	Vγ9Vδ2T	allogeneic Vγ9Vδ2T	—	HCC	Phase 2	NCT02425735
Fuda Cancer Hospital	Vγ9Vδ2T	allogeneic Vγ9Vδ2T	—	TNBC	Phase 2	NCT02418481
H Lee Moffitt Cancer Center and Research Institute	γδ T infusion	APC-expanded donor T-cells administered as a single infusion after an alloHCT	—	AML	Phase 1	NCT05015426
Institute of Hematology & Blood Diseases Hospital	Ex-vivo expanded allogeneic γδT cells	ex-vivo expanded allogeneic γδT cells obtained from a blood-related donor	—	B-NHL, PTCL	Early Phase 1	NCT04696705

Abbreviations: ALL, acute lymphoblastic leukemia; CLL, chronic lymphocytic leukemia; MM, multiple myeloma; AML, Acute myeloid leukemia; mCRPC, Metastatic castration-resistant prostate cancer; RCC, renal cell carcinoma; NSCLC, non-small-cell lung cancer; B-NHL, B-cell non-Hodgkin lymphoma; HCC, Hepatocellular Carcinoma; TNBC, triple-negative breast cancer; PTCL, peripheral T-cell lymphoma.

The Dutch biotech startup Lava Therapeutics have described a humanized bispecific γδ T cell binding antibody (γδ bsTCE). γδ bsTCE directly induces the effective killing of tumor cells through its unique targeting of Vγ9Vδ2 T cells and tumor-associated antigens (TAA). Two of its company’s projects, LAVA-051, and LAVA-1207, have entered clinical phase 2 trials. Multiple myeloma, chronic lymphocytic leukemia, and acute myeloid leukemia all include the antigen CD1d, which is recruited by LAVA-051 to γδ T cells (137). LAVA-051 has been given orphan drug status by the FDA for the treatment of (CLL) based on preliminary data from the Phase 1/2a clinical study and has a satisfactory safety and tolerability profile. Meanwhile, LAVA-1207 was designed to be a γδ bsTCE targeting prostate-specific membrane antigen (PSMA), with its clinical study focusing on metastatic castration-resistant prostate cancer.US-based biotech company IN8bio has also updated positive data from its ongoing phase 1 clinical trial of the allogeneic γδ T cell therapy INB-100 in high-risk AML patients who have previously undergone haploidentical hematopoietic stem cell transplantation (HSCT). From the data, all three patients treated with INB-100 received at least 12 months of follow-up which showed all three were in complete remission (CR). Remarkably, 100% of evaluable-dose patients remained on study and were in CR, with one patient having a progression-free disease course of more than 3 years (NCT03533816).Another ongoing project, INB-200, uses genetically modified autologous γδ T cell immunotherapy for the treatment of glioblastoma (GBM). Data according to the Phase 1 clinical trial of INB-200 for GBM showed that 100% of the six treated patients exceeded the median and expected progression-free survival (PFS). Two of the patients had exceeded the expected overall survival (OS), and the medication was generally well-tolerated and robust.

Innovative developments in CAR-γδ T-cell therapy is also advancing at a rapid pace. The UK company TC Biopharm is developing a new CAR-T therapy that takes advantage of the inherent specificity of γδT cells for phosphorylated antigens expressed only by cancerous and infected cells to develop the ImmuniCAR. OmnImmune, is being tested in a Phase 2b/3 clinical trial, following a 50% CR in Phase 1b/2a clinical data for this therapeutic candidate for AML. Concurrently, Adicet Bio announced clinical data for its allogeneic CAR-γδ T cell therapy ADI-001 for relapsed or refractory B-cell non-Hodgkin lymphoma (NHL). Data from the study showed ADI-001 demonstrated a 75% overall remission rate (ORR) and CR in eight patients who had received multiple prior therapies, including those who relapsed after using CAR-αβ T therapy. Gadeta, a Dutch company, has also innovated in CAR-γδ T-cell therapy, designed to use αβ T cells to carry the T-cell receptor for γδ T cells. The company’s TEGs technology enables the efficient expression of γδ TCR in αβ T cells, mediates tumor-specific proliferation of αβ T cells, and extensively infiltrates CD8^+^ effector T cells and CD4^+^ helper αβ T cells into tumors while not affecting normal organs.

Beyond the aforementioned therapeutic strategies, innovative γδ T cell-based treatments for diverse cancers are continually emerging. Induced pluripotent stem cells (iPSCs) termed T-iPSCs were formed by Watanabe et al. by rearranging the TCR γ chain (Vγ9) and TCR δ chain (Vδ2) gene regions (γδ T-iPSCs). Notably, these γδ T-iPSCs can differentiate into hematopoietic progenitor cells, which could theoretically provide a more potent collection of cells for new cancer research and a nearly infinite source of regenerating cells (138). Similarly, Zeng et al. successfully reprogrammed the γδ T-iPSC line of Vγ9Vδ2 T cells and these cells were modified into NK-like γδ T cells, termed “γδ natural killer T” (γδ NKT) cells (139).

## 7 Limitations and potential of γδ T-cell therapy

It should be emphasized that γδ T-cell therapy still has some issues that need to be addressed. Firstly, the scarcity and low efficiency of *in vitro* expansion remains a serious limitation to entry of γδ T cells into the clinical pipeline. Expanding a considerable number of cell products through *in vitro* methods is crucial for the success of γδ T cell adoptive cell therapy. However, the effectiveness of this approach is limited by the inherent differences between donors (140). Recent research has shown that the level of physical activity in a donor can be used as a gauge for determining the *in vitro* expansion potential of their γδ T cells (124). The dominant subtype of γδ T cells in the peripheral blood of humans and other primates is Vγ9Vδ2 T cells, which account for only 1-10% of circulating lymphocytes in peripheral blood (141, 142). Currently, γδ T cells are largely obtained from peripheral blood mononuclear cells (PBMC) or umbilical cord blood isolated from healthy donors, followed by *in vitro* stimulation and expansion using synthetic PAgs or bisphosphonates (143–148). Gene modification and iPSCs techniques to produce specific γδ T cells in large quantities are major approaches of pharmaceutical companies to improve production and create a more clinically viable option (149). Efforts are underway to identify strategies that amplify the potency of γδ T cells in antitumor activities. For instance, IL-15 which can render a more active phenotype, greater proliferative capacity, and greater cytotoxicity in γδ T cells, is being investigated Combining IL-15 and γδ T cell immunotherapy may be able to enhance antitumor immunotherapy (150). In this regard, more research is warranted to examine the impact of diverse settings on the expansion of γδ T cells *in vitro* and to identify measures to promote the toxicity of Vγ9Vδ2 T cells, including candidates IL-2, IL-15, vitamin C, and TGF-β (126, 151, 152).

Another significant hurdle in advancing γδ T cell therapies pertains to the engineering of γδ T cells (153). For immune cell engineering, the most common method is to use lentivirus or retrovirus transfection. However, compared with ordinary αβ T cells, due to the natural antiviral properties of γδ T cells, viral transfection of γδ T cells is extremely difficult. It is also prone to the loss of CAR genes in cells during culture (115).

The broad spectrum of γδ T cells has to be taken into account when talking about the potential of γδT cell therapy. The heterogeneity of γδ T cells we described previously includes different subpopulations that mediate opposite immune responses to tumors. These subgroups are widely distributed throughout the body (12, 16). In addition to the Vδ2 T cell subpopulation, which is primarily present in peripheral blood and has been developed for antitumor therapy, the Vδ1 T cell subpopulation, which is present in tissues, has demonstrated strong cytotoxic potential against tumors when isolated from a variety of human solid tumors, which may partially address the limitations of current CAR-T therapies against solid tumors (37, 122). Combining contemporary high-throughput technologies to grasp the different subsets of γδ T cells at the single-cell level, such as Vδ1T cells (16), with manipulations such as gene editing techniques to enhance the immunological anti-tumor function of γδ T cells, may increase their potential application.It’s imperative to recognize that the tumor microenvironment is replete with various inhibitory immune cell populations. Immunosuppressive cytokines released by these cells can cause γδ T cells to become pro-tumor oriented and secrete IL-17, which drives cancer progression. In certain instances, the leukemic microenvironment adopts strategies to evade the anti-tumor response of these lymphocytes, leading to their exhaustion or polarization into a tumor-promoting phenotype (18). Confronted with these challenges, targeted screening of anti-tumor subsets, exclusion of pro-tumor subsets, determination of how to prevent the initial tumor killer cells from metamorphosis to promote tumor progression cells, or effective depletion of specific pro-tumor γδ T cell subsets, will be the focus of future research.Much work remains, particularly with regards to dissecting the multitude of subsets present in the body and determining how best to promote their anti-tumor activity. Current production bottlenecks further restrict their clinical application. Nevertheless, with ongoing research, it is anticipated that γδ T cells will cement their place as a cornerstone of cancer immunotherapy in the coming years.

## Author contributions

WY: Writing – original draft. LD: Writing – review & editing. NL: Writing – review & editing. YHW: Writing – review & editing. YFW: Supervision, Writing – review & editing. PW: Funding acquisition, Supervision, Writing – review & editing.
